# Supplementary material for: High-Throughput Profiling of Bacterial Respiration Using the Resipher Reveals Functional Responses to Nutrients and Antibiotics
Source: bioRxiv. 2026 Jun 17:2026.06.17.732880. Preprint. [Version 1] doi: 10.64898/2026.06.17.732880 (PMC13307942; doi:10.64898/2026.06.17.732880)
Supplement: Supplement 1 [file NIHPP2026.06.17.732880v1-supplement-1.pdf]

bioRxiv preprint doi: https://doi.org/10.64898/2026.06.17.732880; this version posted June 17, 2026. The copyright holder for this preprint (which was not certified by peer review) is the author/funder, who has granted bioRxiv a license to display the preprint in perpetuity. It is made available under a CC-BY 4.0 International license.

SUPPLEMENTARY FIGURES AND TABLE

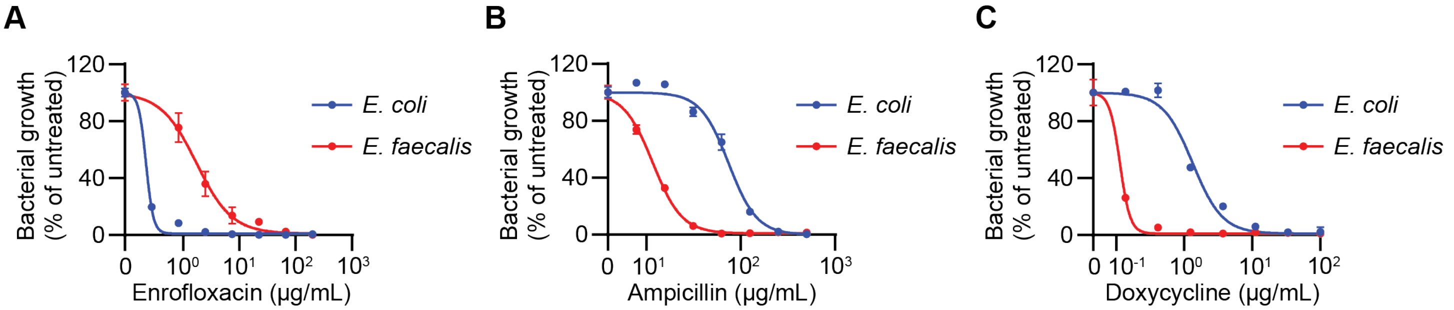

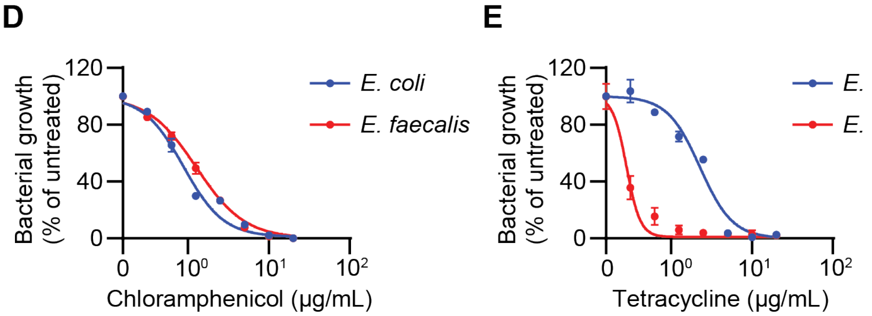

F

	IC_{90} (μg/mL)	
	E. coli	*E. faecalis*
Enrofloxacin	0.3	8
Ampicillin	150	25
Doxycycline	5	0.2
Chloramphenicol	3	6
Tetracycline	6	0.5

Figure S1. Sensitivity of *E. coli* and *E. faecalis* to antibiotics. Growth of *E. coli* and *E. faecalis* in CDM + 1% glucose after treatment with **(A)** enrofloxacin, **(B)** ampicillin, **(C)** doxycycline, **(D)** chloramphenicol and **(E)** tetracycline. Non-linear regression curves using a four-parameter variable-slope model were plotted to determine the inhibitory concentrations of each antibiotic against both bacterial strains. Data are representative of three biological replicates, each containing three technical replicates. **(F)** Calculated IC_{90} concentrations of the antibiotics in (A-E) for *E. coli and E. faecalis*.

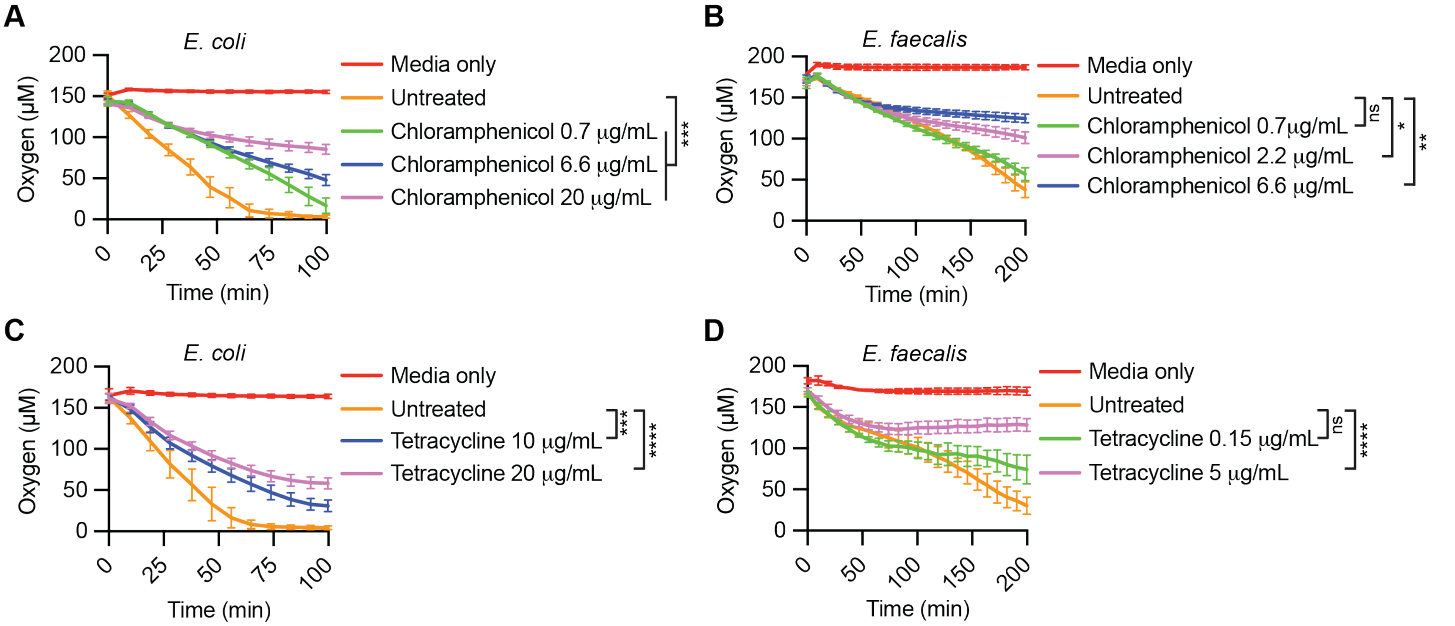

Figure S2. Dose-dependent changes in oxygen consumption in response to bacteriostatic antibiotics. Oxygen consumption measurements of **(A, C)** *E. coli* and **(B, D)** *E. faecalis* in chemically defined media (CDM) + 1% glucose following treatment with **(A, B)** chloramphenicol and **(C, D)** tetracycline at the indicated concentrations. Data shown are a single representative example of three biological replicates and are composed of 6 technical replicates each. Two-way ANOVA statistical tests with Bonferroni's multiple comparisons were performed to assess differences between treated and untreated conditions. (* $P < 0.05$, ** $P < 0.01$, *** $P < 0.001$, **** $P < 0.0001$)

bioRxiv preprint doi: https://doi.org/10.64898/2026.06.17.732880; this version posted June 17, 2026. The copyright holder for this preprint (which was not certified by peer review) is the author/funder, who has granted bioRxiv a license to display the preprint in perpetuity. It is made available under a CC-BY 4.0 International license.

Table S1. Comparison of platforms commonly used to measure oxygen consumption

Feature	Resipher (Lucid Scientific)	O2k (Oroboros)	Seahorse XF (Agilent)
Measurement principle	Fluorescence-based oxygen sensing lid	Advanced Clark-type polarographic oxygen electrode	Fluorescence-based
Culture environment	96-well plates with O_2-sensing lid	Sealed respiratory chamber	Transient microchambers formed during measurement
Time resolution	Continuous monitoring	High temporal resolution (seconds)	Intermittent measurements during assay
Throughput	High (multiple 96-well plates, parallel measurements)	Low (1-2 samples per run)	High (24 or 96 wells)
Sample handling	Standard culturing, minimal perturbation, incubator based	Manual chamber loading and calibration	Capable of media exchange and reagent injection
O_2 consumption by detector	No	Yes	No
Standard culture vessels	Yes	No	No (specialized assay plates required)
Recalibration required / drift issues	Minimal recalibration needed	Requires careful calibration, electrode drift possible	Stable optical system
Cost considerations	Moderate platform and consumable cost)	Moderate (high instrumentation & labor)	High instrument & consumable cost
Key limitation / summary	No rapid injection kinetics during run	Low throughput, labor intensive, not suitable for long-term continuous monitoring	Short duration assays, high cost, technical expertise required
